# Blocking Opioid Receptors in a Songbird Cortical Region Modulates the Acoustic Features and Levels of Female-Directed Singing

**DOI:** 10.3389/fnins.2020.554094

**Published:** 2020-09-17

**Authors:** Sandeep Kumar, Alok Nath Mohapatra, Arvind Singh Pundir, Mukta Kumari, Uzma Din, Sudha Sharma, Atanu Datta, Vasav Arora, Soumya Iyengar

**Affiliations:** National Brain Research Centre, Manesar, India

**Keywords:** cortico-basal ganglia loops, opioid receptors, LMAN, songbirds, cortex, acoustic features

## Abstract

The organization of the anterior forebrain pathway (AFP) of songbirds important for context-dependent singing is similar to that of cortical basal ganglia loops (CBG) in mammals, which underlie motor behaviors including vocalization. Since different components of the AFP express high levels of μ-opioid receptors (μ-ORs) as do CBG loops, songbirds act as model systems to study the role of opioid modulation on vocalization and the motivation to sing. The AFP in songbirds includes the cortical/pallial region LMAN (lateral magnocellular nucleus of the anterior nidopallium) which projects to Area X, a nucleus of the avian basal ganglia. In the present study, microdialysis was used to infuse different doses of the opioid antagonist naloxone in LMAN of adult male zebra finches. Whereas all doses of naloxone led to significant decreases in the number of FD (female-directed) songs, only 100 and 200 ng/ml of naloxone affected their acoustic properties. The decrease in FD song was not accompanied by changes in levels of attention toward females or those of neurotransmitters (dopamine, glutamate, and GABA) in LMAN. An earlier study had shown that similar manipulations in Area X did not lead to alterations in the number of FD songs but had significantly greater effects on their acoustic properties. Taken together, our results suggest that there are reciprocal effects of OR modulation on cortical and basal ganglia components of the AFP in songbirds.

## Introduction

The cortico-basal ganglia (CBG) circuitry is known to be involved in many cognitive functions such as motivation, reward-based learning and the execution of motor functions including speech and vocalization (Doupe and Kuhl, 1999; Kao et al., 2005; Van Lancker Sidtis et al., 2006; Ziegler and Ackermann, 2017). Although speech deficits such as slurring or stuttering are amongst some of the common symptoms of drug addiction (Wasserman and Yahr, 1980), changes in the acoustic properties of speech such as a decrease in pitch, and increased hoarseness (Mirkov and Mitrović, 2019) have not been studied extensively. Furthermore, despite the fact that opioid receptors are expressed in many cortical areas (Bodnar, 2018; van Steenbergen et al., 2019) which project directly to the basal ganglia, the effects of opioid neuromodulation in the context of vocalization has not been explored in detail.

**FIGURE 1:**
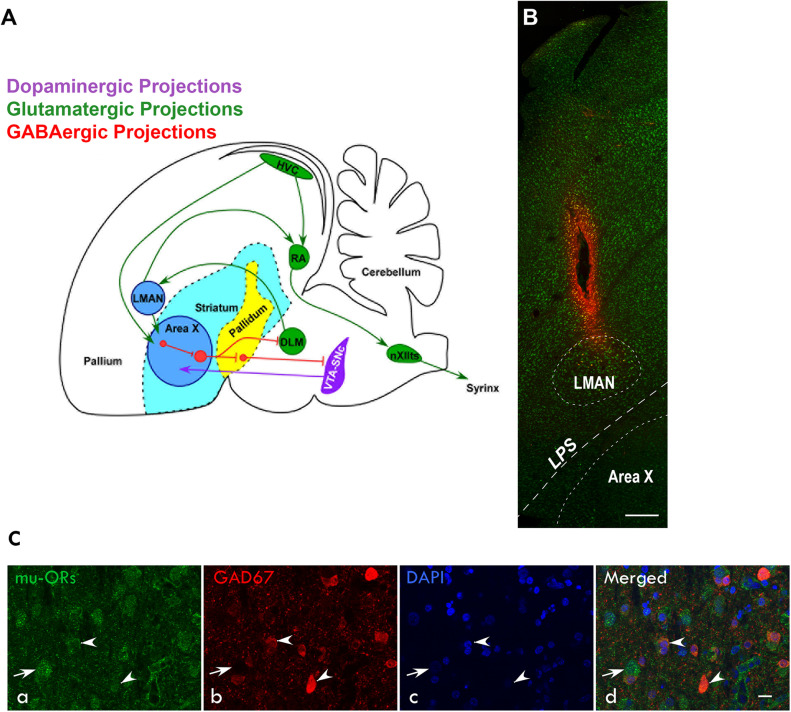
**(A)** Schematic of the song control system and its connections [adapted from Nottebohm et al. (1976); Farries et al. (2005); Gale et al. (2008); Kumar et al. (2019))] showing components of the vocal motor pathway (VMP; HVC→RA→nXIIts, in green) and the anterior forebrain pathway (AFP; LMAN→Area X→DLM→LMAN, blue). Of these regions, HVC, RA, and LMAN are located in the avian pallium (cortex). The AFP connects to VMP via projections from LMAN to RA and connections from HVC to Area X. HVC, cortical premotor nucleus (abbreviation used as a formal name); RA, robust nucleus of the arcopallium; DLM, dorsolateral nucleus of medial thalamus; LMAN, lateral magnocellular nucleus of the anterior nidopallium; Area X, a nucleus of the avian basal ganglia; and nXIIts, tracheosyringeal part of the hypoglossal nucleus. **(B)** Sagittal section of the anterior forebrain stained with green fluorescent Nissl (N21480, ThermoFisher) showing the track and spread of the fluorescent dye micro-Ruby made by the microdialysis probe inserted into LMAN. Dorsal is up and rostral is to the left; scale bar, 250 μm. **(C)** Immunolabeling for μ-ORs and GAD67 in LMAN (a) Both magnocellular projection neurons (*arrow*) and smaller neurons (arrowheads), probably interneurons, are labeled with an antibody against μ-ORs. (b) The magnocellular neurons are not positive for GAD67 whereas the smaller neurons are positive for GAD67. (c) Neuronal nuclei were labeled with DAPI. (d) The merged image shows that smaller neurons co-express μ-ORs and GAD67 and were GABAergic interneurons whereas the magnocellular neurons which are putative excitatory neurons do not express GAD67. Scale bar, 20 μm.

The song control system (SCS, Nottebohm et al., 1976; Figure 1A), a specialized set of neural circuits in songbirds such as zebra finches (*Taeniopygia guttata*), has been utilized extensively to understand mechanisms underlying vocal learning and vocalization (Simpson and Vicario, 1990; Brainard and Doupe, 2000; Bolhuis et al., 2010). Of these circuits, the anterior forebrain pathway (AFP) has been studied in detail for its role in song learning and context-dependent singing in adulthood (Jarvis et al., 1998; Kao and Brainard, 2006) and is strikingly similar to the mammalian CBG (Farries and Perkel, 2002; Carrillo and Doupe, 2004; Gale et al., 2008; Person et al., 2008; Kumar and Iyengar, 2018). It consists of the cortical/pallial region LMAN (lateral magnocellular nucleus of the anterior nidopallium) which projects to Area X (a basal ganglia homologue), (Nixdorf-Bergweiler et al., 1995; Luo et al., 2001). Area X projects to the thalamic nucleus DLM (medial dorsolateral nucleus of the thalamus), which in turn projects to LMAN, completing the CBG loop (Bottjer et al., 1989; Luo and Perkel, 1999). Whereas Area X receives projections from a specific subset of neurons in the pallial nucleus HVC (Nottebohm et al., 1982; Dutar et al., 1998), another set of HVC neurons project to the premotor nucleus RA (robust nucleus of the arcopallium), (Bottjer et al., 1989; Fortune and Margoliash, 1995), forming the vocal motor pathway (VMP) which innervates the syrinx or vocal organ (Nottebohm et al., 1976; Wild, 1993).

An earlier study from our lab showed that all components of the AFP and VMP in zebra finches express both μ- and δ-opioid receptors (μ-and δ-ORs, respectively), although levels of μ-ORs were significantly higher than those of δ-ORs (Khurshid et al., 2009). Whereas μ-OR signaling underlies reward associated with natural stimuli (such as birdsong), δ-ORs are known to be anxiolytic and are associated with positive affect (Lutz and Kieffer, 2013; Valentino and Volkow, 2018). Furthermore, systemic injections of the OR antagonist naloxone decreased the motivation of adult males to sing courtship (female-directed; FD) songs and also affected their acoustic properties (Khurshid et al., 2010). Presumably, these effects would have resulted from blocking both μ-and δ-ORs, since naloxone binds with the highest affinity to μ-ORs and to a lesser extent to δ-ORs (Magnan et al., 1982; Deviche, 1997). However, site-specific infusions of naloxone in Area X (Kumar et al., 2019) did not lead to changes in the number of FD songs, implying that other regions of AFP or a different region of the brain altogether might have led to these changes. Indeed, (Kelm-Nelson et al., 2013) demonstrated that infusing naloxone in the medial preoptic area (mPOA) of other songbirds (starlings, *Sturnus vulgaris*) which were good singers (that is, sang an average of 7.44 ± 4.53 FD songs during a 20-min period) led to a decrease in their rates of singing.

Since we had found a number of changes in the spectro-temporal features of motifs as well as in individual syllables of FD song when naloxone was infused directly into Area X (Kumar et al., 2019), we were curious to know whether the results would be similar when opioid receptors were blocked specifically in LMAN, which projects directly to Area X (Nixdorf-Bergweiler et al., 1995; Luo et al., 2001), and RA (Bottjer et al., 1989; Mooney, 1992). A number of studies have demonstrated the role of LMAN in regulating variability in songs learned during the sensitive period (Bottjer et al., 1984) as well as during social-context dependent singing in adults (Kao and Brainard, 2006; Kao et al., 2008; Hampton et al., 2009; Stepanek and Doupe, 2010). Microstimulating LMAN immediately altered the fundamental frequency and amplitude of individual syllables while birds sang (Hamaguchi and Mooney, 2012; Kojima et al., 2018). Furthermore, increasing protein levels of NR2B (N-methyl-D-aspartate or NMDA subtype B) receptors in LMAN of adult male zebra finches which may have led to changes in its activity (Chakraborty et al., 2017) led to increases in the number of introductory notes and syllables, song duration, pitch and variability in the sequence of songs. In contrast, inactivating LMAN led to a decrease in variability of the acoustic properties of songs (Stepanek and Doupe, 2010), indicating that it is important for generating variability in birdsong. Other studies (Kojima et al., 2018) have also demonstrated that rapid within-syllable variability is relayed from Area X via its projections to DLM, which in turn projects to LMAN.

In order to study the effects of blocking OR modulation in LMAN, we specifically infused the opioid antagonist naloxone in this region while adult male birds sang to females. To our surprise, infusions of naloxone in LMAN led to significant decreases in FD singing, suggesting that it may play a role in the motivation to sing. We also found that these manipulations altered the acoustic features of motifs and individual syllables, which were different from those produced by blocking ORs in Area X (Kumar et al., 2019).

## Materials and Methods

All experimental procedures were approved by the Institutional Animal Ethics Committee at the National Brain Research Centre, Manesar (NBRC), India which were in compliance with the Committee for the Purpose of Control and Supervision of Experiments on Animals (CPCSEA), India. A total of 9 adult male zebra finches (>120 days) housed in the aviaries at the NBRC Animal House were used for these experiments. Whereas naloxone was infused in LMAN in 6 birds, the fluorescent tracer FluoroRuby was injected in Area X of 3 birds to confirm whether the magnocellular projection neurons in LMAN also expressed μ-ORs (see below). A group of 3–4 female birds was used to elicit courtship songs from the males. Birds were allowed *ad libitum* access to food and water and maintained at a 12/12-h light and dark cycle.

### Surgical Procedures

Birds (*n* = 6) were anesthetized using intramuscular (I.M.) injections of ketamine [25 mg/kg body weight (bw)], xylazine (2.5 mg/kg bw), and diazepam (8 mg/kg bw; Kojima and Doupe, 2007; Pytte et al., 2010; Prather, 2012; Kumar et al., 2019) also containing the analgesic Butorphanol tartrate (Butrum, 0.1 mg/kg bw). A tungsten electrode (1 MΩ at 1 kHz, Microprobe Inc., MD, United States) was lowered into the brain through a burr hole drilled in the skull over the left hemisphere 4.7 mm rostral and 1.6 mm lateral to the bifurcation of the superior sagittal sinus (the “Y” point). The boundaries of LMAN were determined by observing spontaneous activity in response to playbacks of the bird’s own songs [BOS, (Doupe, 1997)], recorded prior to the surgery (Figures 1A,B). A guide cannula (CMA 7, Microdialysis, Solna, Sweden) was implanted into the brain at the dorsal or anterior boundary of LMAN at a depth of 1.2–2.1 mm (Nixdorf-Bergweiler and Bischof, 2007; Stepanek and Doupe, 2010) and fixed with cyanoacrylate glue and epoxy (cf. Kumar et al., 2019). After surgery, birds were administered intramuscular injections of Flumazenil (0.3 mg/kg bw; F6300, Sigma-Aldrich) to block the effects of diazepam and kept near a heat source to help them recover rapidly from anesthesia (Prather, 2012). For the next 3 days, birds were injected with the analgesic (Butrum 0.1 mg/kg bw) and also provided with the oral antibiotic Cephalexin (35 mg/kg bw; Phexin Dry Syrup, GlaxoSmithKline Pharmaceuticals Limited).

#### FluoroRuby Injections in Area X to Label LMAN Projection Neurons

Burr holes were drilled in the skull bilaterally (4.7 mm rostral and 4 mm lateral to the “Y” point) of 3 birds. A glass micropipette (outer diameter of tip ∼50–75 μm) was filled with the fluorescent dye FluoroRuby [1.5% dissolved in aCSF (artificial cerebrospinal fluid), ThermoFisher, D1817] and attached to a microinjection system [Nanoliter 2010 W/Micro (World Precision Instruments, United States) controlled by a Microsyringe pump controller (Model: Micro4, WPI, Microsyringe)]. The micropipette was inserted at an angle of 45° at a depth of 3.5 mm so as to reach Area X while avoiding LMAN which lies just above it. Two injections of FluoroRuby (100 nl each, at the rate of 5 nl/s) were made at two locations (A-P coordinates 4.6 mm and 4.8 mm) to target Area X.

### Microdialysis of Naloxone in LMAN

Microdialysis experiments were carried out using procedures described in Kumar et al. (2019), which was adapted from Sasaki et al. (2006). Briefly, a microdialysis probe (CMA-7, Cuprophane membrane length 1 mm, CMA Microdialysis) was used to infuse aCSF (pH 6; composition: NaCl 147 mM/L, KCl 2.7 mM/L, CaCl_2_ 1.2 mM/L, and MgCl_2_ 0.85 mM/L) or the drug (naloxone, N7758; Sigma-Aldrich) dissolved in aCSF using a syringe pump (CMA 402, CMA Microdialysis) in LMAN. The probe was attached to the guide cannula via a swivel suspended from a Multi-Axis Counter-Balanced Lever Arm (375/D/22 Dual channel swivel, 22 ga and SMCLA, 9 cm; Instech Laboratories, Inc., Plymouth, United Kingdom). To acclimatize experimental birds to the microdialysis probe, it was inserted into the guide cannula the night before the experiment after flushing it with aCSF. During the experiments, dialysates were collected in the dark on ice in an Eppendorf tube containing 1 mM ascorbic acid (05878, Sigma-Aldrich, United States) 15% of the total dialysate volume to minimize oxidative degradation of dopamine (DA). Dialysate samples were lyophilized and stored at −80°C until they were analyzed. After collecting dialysates for different doses of naloxone, the fluorescent dye micro-Ruby (2% dissolved in aCSF, D7162, Molecular Probes, United States) was infused via the microdialysis probe to gauge the spread of naloxone into LMAN (Figure 1B).

### Behavioral Experiments

**FIGURE 2:**
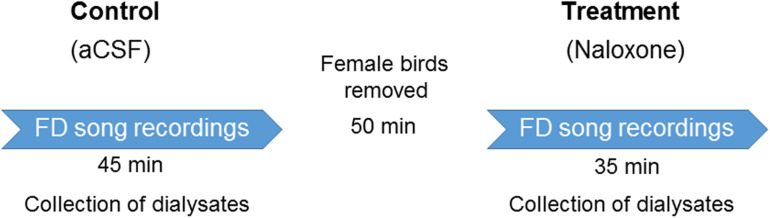
Experimental design. After recovering from surgeries for cannula implantation in LMAN, adult male zebra finches (*n* = 6) were infused with aCSF and female-directed (FD) songs were recorded for 45 min. During this period, the dialysate containing extracellular fluid was collected from LMAN. At the end of these recordings, the cage containing females was removed to ensure auditory and visual isolation from the males for a period of 50 min. This was followed by infusions of any of the doses of naloxone (50; 100; or 200 ng/ml). During this period, FD songs were recorded and dialysate from LMAN was collected. A day’s gap was given before infusing any of the doses of naloxone to ensure that it was completely washed out of the brain. Each dose of naloxone was administered to each bird 3–4 times during the course of the experiment and the values for behavior and neurotransmitter levels were collated for each bird.

Experiments were performed in a custom-made sound-attenuated box 4–7 days after surgery, when singing returned to baseline levels. All experiments were conducted in the morning within 1 h of turning on the lights in the room where the birds were housed. A set of 3–4 female birds were placed in a cage opposite experimental birds to elicit FD singing (Khurshid et al., 2010). The same set of females was used for control and drug infusions in males on the same day. Before the experiments, aCSF was infused at the rate of 3 μl/min for 10–15 min to let the flow rate stabilize. Dialysates were collected for 45 min during aCSF infusions and for 35 min during naloxone infusion separated by 50 min of auditory and visual isolation from the females. Experiments for each dose (aCSF and naloxone infusion) were repeated for 3–4 days with a day’s gap in between (Kumar et al., 2019; Figure 2). All audio recordings were obtained with a microphone (Sennheiser e614) connected to a computer via an M-Track Quad device (M-Audio) and the SA+ recorder (Sound Analysis Pro software version 1.02, Tchernichovski et al., 2000). Male birds’ behavior was recorded with a webcam (Logitech HD C210). Only those FD motifs/songs were analyzed in which males sang near females (either facing them or sideways), which occurs in natural courtship.

### Estimation of Neurotransmitters by LC-MS/MS

Estimation of neurotransmitters was performed by UPLC-MS/MS at a commercial facility (Sandor Life Sciences Pvt. Ltd. Hyderabad) modified from González et al. (2011); Kumar et al. (2019). As a result of technical issues with one of the samples, we analyzed samples from 5 birds of the 6 used for these experiments. Briefly, a SYNAPT G2 QTOF (Waters, United Kingdom) mass spectrometer equipped with an electrospray ionization (ESI) source was used in the positive mode. The desolvation temperature was set at 350°C, source temperature at 150°C and the source gas flow at 30 ml/min. Cone gas flow was kept at 30 L/h and desolvation gas flow at 80 L/h. The capillary voltage was set at 3.5 kV and the cone voltage at 40 V. The sampling cone and extraction cone voltage were kept at 45 kV and 4.5 kV, respectively. The column temperature was maintained at 40°C. The acquisition was performed in the positive resolution mode with an acquisition time of 15 min. The data was collected between a range of 50–500 Da with a scan time of 0.5 s in the continuum format. The collision energy was as follows: Function-1 Low Energy – Trap collision energy and transfer collision energy (On – 6 V); Function-2 High Energy – Ramp Trap Collision Energy (On – 20 V to 45 V), Ramp Transfer Collision Energy (Off).

Liquid chromatography was performed on an ACQUITY UPLC system (Waters, United Kingdom). The separation of all samples was performed on a Union column maintained at 40°C. A gradient elution program prepared on Masslynx V4.1 software (Waters, United Kingdom) was used for the chromatographic separation (15 min, flow rate: 0.5 ml/min) with the mobile phase A (0.1% formic acid in water), and mobile phase B (0.1% formic acid in acetonitrile). The gradient run was started with 98% of A, then shifted to 20% in the 10th min. In the next 2 min, 20% of A was shifted to 100% of B. The first line condition was achieved in the next 3 min. Standards were prepared in 0.1% formic acid and 15% 1 mM ascorbic acid in Milli-Q water. Lyophilized dialysate samples were dissolved in the same solvent and a volume of 10 μl was injected at a flow rate of 0.3 μl/min for the analysis.

### Validation of Antibodies Directed Against μ-ORs and GAD67 (Glutamic Acid Decarboxylase)

The antibody used to detect μ-ORs (Abcam, ab10275, made in rabbit) has been validated using western blots in an earlier publication from our lab (Kumar et al., 2019). To validate the specificity of the anti-GAD67 antibody (Abcam, ab26116, raised in mouse), a western blot was performed by the same method using whole brain protein lysate from adult male zebra finches separated by SDS-PAGE (11%; Kumar et al., 2019; Sen et al., 2019). The anti-GAD67 antibody was raised against a synthetic peptide corresponding to amino acids 87–106 of Human GAD67. This sequence has 95% identity with the GAD67 sequence of zebra finches (XP_002198534.2).

Bands in the range of 180 and 140 kDa, corresponding to trimers and dimers of GAD67 either with itself or with GAD65 (Kanaani et al., 1999) were observed, since only 3% of the total GAD67 in the brain exists as monomers (Sheikh and Martin, 1996; Supplementary Figure 1). Although we tried varying concentrations of beta-mercaptoethanol for denaturation of the protein lysate, the GAD67 dimers were not broken down into monomers (∼67 kDa) since they may not require the formation of disulfide linkages (Battaglioli et al., 2005). Smaller fractions in the range of 110 and 50 kDa reported (Benitez et al., 2014; Yu et al., 2016) in the rat brain using the same antibody (ab26116, Abcam) were also present in our western blots. Furthermore, a prominent band was observed in the 31 kDa range, representing a truncated form of GAD67 observed in the mouse brain (Szabo et al., 1994; Battaglioli et al., 2005; Sha et al., 2005).

### Perfusion and Histology

After experiments, birds were overdosed with ketamine and 2 mg/kg bw of xylazine and perfused transcardially with 0.01M PBS (phosphate-buffered saline, pH7.4), followed by 4% paraformaldehyde containing 0.04% glutaraldehyde. Brains were immersed in 30% sucrose for cryoprotection and 40 μm thick serial sagittal cryosections were cut for Nissl staining and sequential double immunohistochemistry (Kumar et al., 2019). Briefly, sections were incubated in an antigen-unmasking solution (pH 6.0; Vector laboratories, H-3300) at 80°C in a water bath (20 min), followed by quenching in 2% H_2_O_2_ (20 min). Non-specific antigens were blocked with 5% normal goat serum (NGS; Vector laboratories, S-1000) and 1% Bovine serum albumin (BSA; Vector Laboratories, A7906) for 1 h at room temperature followed by incubation in a cocktail of the primary antibodies [anti-μ-OR antibody (1:500), Abcam, ab10275 and anti-GAD67 (1:1000), Abcam, ab26116] made in 1% BSA and 3% NGS at 4°C for 48 h. Sections were then incubated in a solution containing goat anti-mouse secondary antibodies tagged with Alexa Fluor 594 (1:250, ThermoFisher Scientific, A-11005), and goat anti-rabbit secondary antibodies tagged with Alexa Fluor 488 (1:250, ThermoFisher Scientific, A-11008) to visualize GAD67 and μ-ORs, respectively, for 3 h at room temperature. A similar protocol was followed for the immunohistochemical staining of μ-ORs alone, in the sections wherein LMAN projection neurons were retrogradely labeled by FluoroRuby injections in Area X. After every step, sections were rinsed with 0.01M PBS except between blocking and primary antibody incubation. Sections were mounted on subbed slides, cover-slipped using anti-fade nuclear staining medium (DAPI, Vectashield, Vector laboratories, H-1200) and imaged using a Zeiss AxioCam MRm camera attached to a fluorescence microscope (Axioimager Z1, Carl Zeiss, Germany).

### Behavioral Analysis

Numbers of bouts, songs and introductory notes were counted following aCSF or naloxone infusion. The first 5 min of recordings following infusions of aCSF (control) or naloxone were discarded since it took 5 min for the drug to reach the probe from the pump. Not collecting data from this period also excluded the possibility of sampling behavioral changes caused by the stress of handling.

#### Acoustic Properties of Motifs and Individual Syllables

The SA+ software was used to analyze raw data of spectral features [mean FM (frequency modulation), mean pitch, mean pitch goodness, mean entropy, mean AM (amplitude modulation), and mean frequency] and temporal features (syllable duration and intersyllable intervals) of songs [SA+, version 1.02, and (Khurshid et al., 2010)]. Spectro-temporal features of motifs [*n* = 414 (aCSF, 6 birds); *n* = 87 (50 ng/ml naloxone, 6 birds); *n* = 81 (100 ng/ml naloxone; 5 birds), and *n* = 89 (200 ng/ml naloxone, 5 birds)] were used for comparisons before and after infusion of different doses of naloxone. For analysis of changes in the acoustic features of individual syllables, the most frequently occurring syllable types (Zann, 1996; Kumar et al., 2019) in our birds included (i) harmonic stacks [5 birds; *n* = 361 (aCSF); 96 (100 ng/ml naloxone); and 150 (200 ng/ml naloxone)] (ii) frequency-modulated syllables [6 birds; *n* = 907 (aCSF); 260 (100 ng/ml naloxone); and 337 (200 ng/ml naloxone)] (iii) high-pitched syllables [4 birds; *n* = 534 (aCSF); 109 (100 ng/ml naloxone); and 174 (200 ng/ml naloxone)], and (iv) complex syllables which had a high-pitched part immediately followed by a frequency-modulated part [2 birds; *n* = 316 (aCSF); 89 (100 ng/ml naloxone); and 62 (200 ng/ml naloxone)]. Further, only clearly recorded motifs and syllables without cage sounds or female vocalizations in the background were included for analysis using the SA+ software. For segmentation criteria of motifs and syllables in SA+, the “or” combination for Wiener entropy (< -3.6) and amplitude (if more than) were chosen. The amplitude threshold was adjusted at different levels for different birds but was maintained in the range of ± 1 for all groups.

### Statistics

All statistical tests were performed using Sigmaplot 13 (Systat Software, Inc.). Since the number of introductory notes/bouts/motifs varied across birds during different experiments, these parameters were normalized for each bird as a percentage of the control values obtained during aCSF infusion on the same day. Normalized data was pooled and a One Way Repeated Measures Analysis of Variance test (RM ANOVA) was performed, with *P* < 0.05 deemed significant. Raw data obtained from SA+ software was pooled together and the Kruskal–Wallis One Way ANOVA on Ranks was used to compare spectro-temporal features of motifs and syllables during control and naloxone infusions, with *P* values < 0.01 deemed significant and the Dunn’s *post hoc* test was used to analyze the difference amongst groups. The Kruskal-Wallis ANOVA test was used here since the number of motifs/syllables were unequal in each group and their distributions did not pass normality or equal variance tests. The Kruskal-Wallis One Way ANOVA on ranks test was also used to compare the acoustic features of FD motifs sung during infusions of aCSF or naloxone (at different doses) across different days to ensure that there were no major variations in these features over the course of the experiments (Supplementary Table 1). Additionally, the coefficient of variation (CV; standard deviation/mean) for the acoustic features of motifs and individual syllables in songs sung during aCSF or naloxone infusions was compared using RM ANOVA.

Levels of each neurotransmitter (dopamine, glutamate and GABA) collected during naloxone infusion were normalized as a percentage of the levels of these neurotransmitters measured on the same day during aCSF infusions in LMAN. The RM ANOVA test was performed to detect relative changes in each neurotransmitter in response to infusions of different doses of naloxone in LMAN.

## Results

### Magnocellular Projection Neurons as Well as Interneurons in LMAN Expressed μ-ORs

**FIGURE 3:**
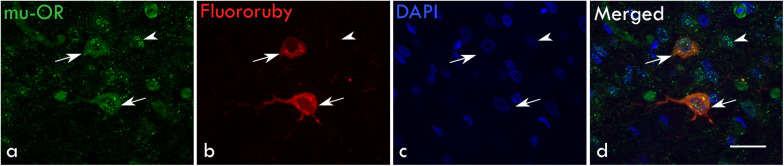
Retrograde labeling for magnocellular projection neurons in LMAN: **(a)** Both magnocellular neurons (*arrows*) and smaller GABAergic interneurons express μ-ORs *(arrowhead*). **(b)** The magnocellular neurons of LMAN (*arrows*) were retrogradely labeled by injections of the dye FluoroRuby in Area X. **(c)** Neuronal nuclei were labeled with DAPI. **(d)** The merged image shows that retrogradely-labeled projection neurons express μ-ORs (*arrows*) and are magnocellular whereas the smaller neurons (*arrowhead*) which have not been back-labeled by FluoroRuby are immunopositive for μ-ORs. Scale bar, 20 μm.

Earlier studies (Nottebohm et al., 1982; Bottjer et al., 1989; Vates and Nottebohm, 1995) had demonstrated that besides projecting to RA, magnocellular neurons in LMAN also project to Area X, which is similar to the cortico-striatal pathway (Figure 1A). Both projection neurons and smaller neurons (interneurons) in LMAN express μ-ORs (Khurshid et al., 2009) which we confirmed in the present study (Figure 1C). To further confirm that magnocellular neurons in LMAN projected to Area X and expressed μ-ORs, we injected the neuroanatomical tract-tracing dye FluoroRuby (ThermoFisher, D1817) in Area X and used immunohistochemistry to detect μ-ORs in coronal brain sections containing both Area X and LMAN. We found that cell bodies of all retrogradely labeled LMAN neurons were magnocellular and positive for μ-ORs (Figure 3).

### Blocking ORs in LMAN Led to a Significant Decrease in Female-Directed Singing

**FIGURE 4:**
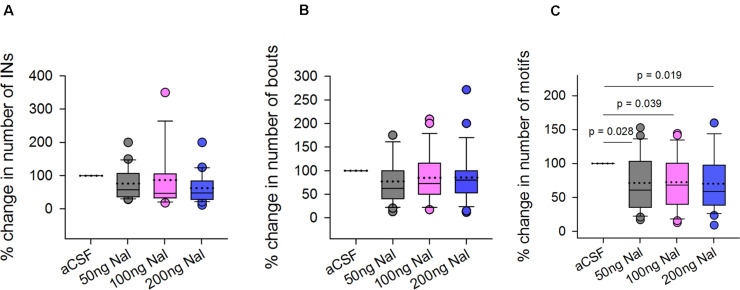
Effects of infusing naloxone in LMAN on different features of FD singing. Statistical comparisons using One Way RM ANOVA demonstrated no significant differences in the **(A)** number of introductory notes and **(B)** bouts following infusions of different doses of naloxone in LMAN versus controls (aCSF infusions). **(C)** All doses of naloxone caused significant decreases in the number of motifs compared to those during infusions of aCSF. Data is expressed as percentage of control values recorded during aCSF infusion. All outliers (colored circles) are shown above and below boxplots and were included in the statistical analysis. The dotted line within the box represents means whereas the solid line represents medians.

To study whether opioid modulation in LMAN had any effect on levels of FD singing, we counted the number of introductory notes (INs), bouts and motifs during naloxone infusion in LMAN. Whereas there was a trend toward a decrease in the number of INs (Mean ± SEM: 76.41 ± 10.48 for 50 ng/ml; 86 ± 22.37 for 100 ng/ml; 62.70 ± 9.93 for 200 ng/ml) and the number of bouts (77.01 ± 9.69 for 50 ng/ml; 84.69 ± 10.78 for 100 ng/ml; 85.37 ± 11.32 for 200 ng/ml naloxone) following naloxone infusion in LMAN versus controls, these differences were not significant (Figures 4A,B, respectively). However, there was a significant decrease in the number of female-directed motifs sung by experimental birds during naloxone infusions versus controls [main effects; One Way RM ANOVA; *F* = 4.28; df = 3; *P* = 0.008; Figure 4C]. The % change in the number of motifs (71.34 ± 8.56, *P* = 0.028 for 50 ng/ml; 72.48 ± 8.20, *P* = 0.039 for 100 ng/ml, and 70.19 ± 8.46, *P* = 0.019 for 200 ng/ml) decreased significantly for all doses of naloxone compared to aCSF infusions used in our study. Furthermore, there were no changes in the number of introductory notes per bout (aCSF: 4.97 ± 0.38, 50 ng/ml naloxone: 4.40 ± 0.43, 100 ng/ml naloxone: 4.31 ± 0.82, and 200 ng/ml naloxone: 5.20 ± 0.66) or that of number of motifs per bout (aCSF: 5.88 ± 0.51, 50 ng/ml naloxone: 4.40 ± 0.33, 100 ng/ml naloxone: 5.34 ± 0.68, and 200 ng/ml naloxone: 5.20 ± 0.49) following naloxone infusion in LMAN.

Taken together, our results suggest that blocking ORs in LMAN (which would disinhibit its projection neurons) leads to a decrease in FD singing compared to controls. Our findings also demonstrate that naloxone infusion in LMAN did not affect the number of introductory notes per bout or number of motifs per bout versus those of controls.

### Visual Attention Toward Females Did Not Change as a Result of Blocking ORs in LMAN

**FIGURE 5:**
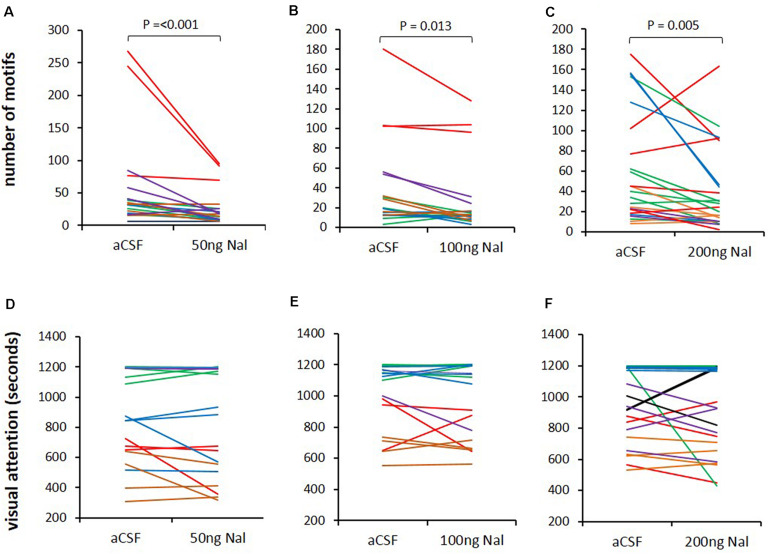
Blocking ORs in LMAN does not affect visual attention toward females. **(A–C)** The number of FD motifs sung during infusions of different doses of naloxone (50, 100, and 200 ng/ml) decreased compared to those sung during aCSF infusion in LMAN. **(D–F)** Visual attention (time spent by males attending to females) was not affected by any dose of naloxone infused into LMAN during the last 20 min of video recordings for each bird. The color of the lines in both graphs (song and visual attention) represents raw data from different recording sessions in each experimental bird analyzed with the paired Rank Sum Test.

Since all three doses of naloxone infused in LMAN led to significant decreases in the number of FD songs, we wanted to study whether this decrease resulted from low levels of the motivation to sing or reduced visual attention toward females. To test this, we measured the time spent by the male looking toward females during aCSF infusion and naloxone infusion on the same day and also counted the number of FD motifs. A paired Rank-Sum Test on the number of motifs and time spent attending to females from the last 20 min of recordings revealed that despite significant decreases in number of motifs for all doses of naloxone compared to controls (50 ng/ml: *Z* = −3.385, *P* = 0.001, *n* = 19; 100 ng/ml: *Z* = −2.439, *P* = 0.013, *n* = 18; 200 ng/ml: *Z* = −2.814, *P* = 0.005, *n* = 23; Figures 5A–C), there were no significant changes in the visual attention paid by males toward females (Time in seconds: 50 ng/ml: 825.26 ± 80.71 versus aCSF: 863.42 ± 69.86; 100 ng/ml: 970.56 ± 56.70 versus aCSF: 990.28 ± 53.48; 200 ng/ml: 911.87 ± 58.16 versus aCSF: 948.30 ± 49.52; Figures 5D–F). These results suggest that blocking μ-ORs in LMAN specifically affects the motivation of males to sing to females without altering overall levels of attention.

### Levels of Neurotransmitters in LMAN Did Not Change Significantly During Naloxone Infusion

**FIGURE 6:**
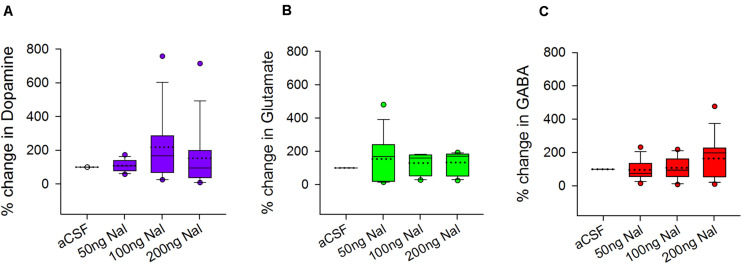
Effects of infusing naloxone on the levels of different neurotransmitters in LMAN. There were no significant changes in the levels of **(A)** dopamine (purple), **(B)** glutamate (green), and **(C)** GABA (red) during infusion of any dose of naloxone in LMAN versus controls. Data is expressed as a percentage of corresponding neurotransmitter levels during aCSF infusion on the same day. Outliers (colored circles) were included in the statistical analysis; in the boxplots, dotted line represents means; solid line represents medians.

Both glutamatergic projection neurons and GABAergic interneurons in LMAN express μ-ORs (Figure 3). Furthermore, LMAN is innervated by excitatory projections from the thalamic nucleus DLM (Bottjer et al., 1989) and low levels of catecholaminergic input (Bottjer, 1993) likely from VTA-SNc (ventral tegmental area-substantia nigra complex). To study whether blocking μ-ORs would affect the levels of neurotransmitters in LMAN, we measured the levels of DA, glutamate (Glut) and GABA in the dialysates containing extracellular fluid collected during infusions of aCSF or naloxone in LMAN. A One Way RM ANOVA revealed that other than a small, non-significant increase in DA (218.18 ± 54.08% mean ± SEM) which was highly variable (median: 167.07%, first and second quartiles: 68.31 and 285.73) following infusions of 100 ng/ml naloxone in LMAN, there were no significant changes in the levels of any of these neurotransmitters in LMAN during naloxone infusions versus controls (DA: *F* = 1.99, df = 3, 35, *P* = 0.130; Figure 6A; Glut: *F* = 1.12, df = 3, 35, *P* = 0.35; Figure 6B; and GABA: *F* = 1.94, df = 3, 35, *P* = 0.14; Figure 6C). It is possible that the lack of significant changes in DA levels in LMAN results from the fact that it does not receive high levels of catecholaminergic innervation in zebra finches (Sakaguchi and Saito, 1989; Bottjer, 1993; Soha et al., 1996; Harding et al., 1998; Singh and Iyengar, 2019).

### Blocking ORs in LMAN Affected the Acoustic Structure of FD Songs

#### Effects on the Acoustic Structure of Motifs

**FIGURE 7:**
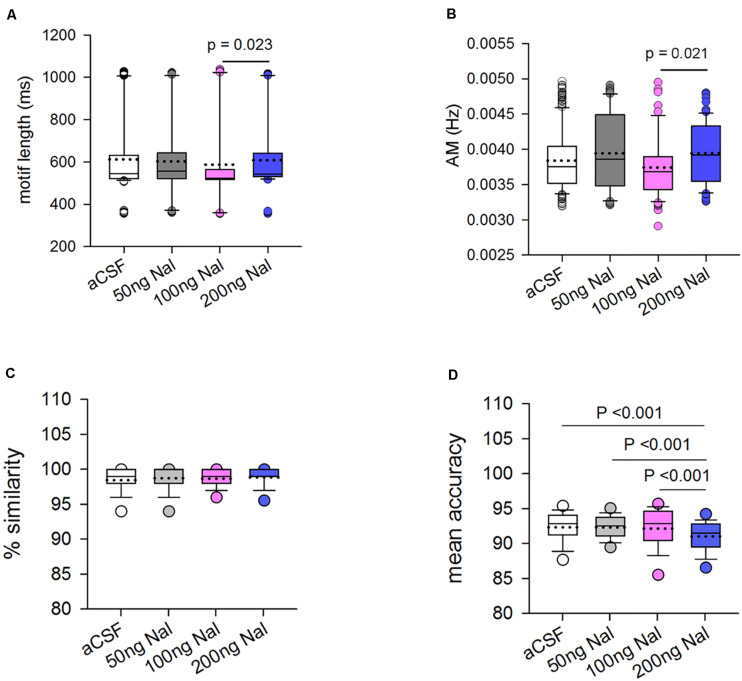
Blocking ORs in LMAN changes some of the acoustic features of FD motifs. **(A)** The motif length decreased significantly during infusions of 100 ng/ml naloxone versus 200 ng/ml naloxone. **(B)** The only spectral feature affected was amplitude modulation (AM) which decreased significantly during infusions of 100 ng/ml naloxone compared to 200 ng/ml naloxone. Changes in % similarity and mean accuracy scores of motifs sung during naloxone infusion in LMAN versus controls. **(C)** Similarity scores for motifs sung during naloxone infusion were calculated by comparing motifs sung during drug infusion to motifs sung during aCSF infusion in LMAN (*n* = 188 for 50 ng/ml naloxone, 106 for 100 ng/ml naloxone, and 130 for 200 ng/ml naloxone). For controls, a self-similarity score was generated by comparing motifs within aCSF to each other in different combinations (*n* = 292). There were no significant differences in similarity at the level of motifs sung when μ-ORs were blocked in LMAN versus those in controls. **(D)** Mean accuracy of songs was significantly lower during infusions of 200 ng/ml naloxone in LMAN versus infusions of aCSF or lower doses of naloxone, suggesting that blocking μ-ORs in LMAN leads to changes in the acoustic structure of individual syllables.

Infusions of naloxone in LMAN led to significant changes in the motif length of FD song (*H* = 22.17, df = 3, *P* = 0.023; ANOVA on ranks, Figure 7A). Dunn’s multiple pairwise comparisons revealed that these differences arose as a result of the reduction in motif length during 100 ng/ml naloxone versus 200 ng/ml naloxone (medians: 523.60 ms versus 543.32 ms; *P* = 0.023). However, none of the doses of naloxone caused significant decreases in motif length compared to motifs sung during aCSF infusions (545.36 ms; Supplementary Table 2). Amongst the spectral features, only amplitude modulation (AM) was affected by infusions of naloxone in LMAN (*H* = 10.01, df = 3, *P* = 0.018, Figure 7B). *Post hoc* tests showed that there was a decrease in AM during infusions of 100ng/ml of naloxone compared to those at 200 ng/ml naloxone (medians: 0.0037 versus 0.0039; *P* = 0.021). Other spectral features were not affected by infusion of any dose of naloxone in LMAN (Supplementary Table 2).

#### Similarity Scores for Motifs Sung During Naloxone Infusion in LMAN

To study whether there were any changes in the acoustic structure of FD songs as a result of blocking ORs in LMAN, similarity scores were generated using the SA+ software by comparing different combinations of motifs sung during infusions of different doses of naloxone and those sung during aCSF infusion (controls). These scores were then compared to self-similarity scores for controls, generated by comparing different combinations of motifs sung during aCSF infusion using One-way ANOVA on ranks. We found that there were no significant differences in % similarity between control and naloxone infusion in LMAN at any dose (Figure 7C). However, one of the measures which contributes in % similarity, that is, mean accuracy, a measure of changes in the acoustic structure of individual syllables, was significantly different when compared across control and treated birds (*H* = 39.85, df = 3, *P* ≤ 0.001; Figure 7D). *Post hoc* tests revealed that there was a significant decrease in the mean accuracy scores during infusions of 200 ng/ml naloxone (*n* = 130; 91.03 ± 0.20) compared to aCSF (*n* = 292; 92.35 ± 0.144, *P* < 0.001) and compared to other doses of naloxone [50 ng/ml (*n* = 188; 92.33 ± 0.14, *P* < 0.001) and 100 ng/ml (*n* = 106; 92.17 ± 0.31, *P* < 0.001); Figure 7D]. These results suggest that the acoustic structure of FD motifs at the level of individual syllables was affected by the highest dose of naloxone used in our study to block ORs in LMAN.

#### Effects of Naloxone Infusion in LMAN on the Variability of Motifs or Syllables in FD Song

An earlier study (Stepanek and Doupe, 2010) had demonstrated that infusions of the GABA(A) receptor agonist muscimol in LMAN inactivated this nucleus and significantly decreased the variability normally present in undirected (UD) songs to the low levels of syllable variability present in FD songs. Although we did not record UD songs in our study following naloxone infusions in LMAN, we decided to study changes in the variability of FD songs sung by experimental birds since blocking the inhibitory ORs expressed by LMAN neurons (Khurshid et al., 2009) would lead to an increase in its activation. As explained earlier, self-similarity scores were generated using the SA+ software for motifs sung during infusions of 50, 100, and 200 ng/ml naloxone or motifs sung during aCSF infusion. By comparing these measures (using ANOVA on ranks, Supplementary Figures 2A,B), we found no significant differences, suggesting that the variability of motifs in FD song sung while ORs were blocked in LMAN was not affected compared to controls. An analysis of the coefficient of variation (CV) of various spectral features of motifs sung during naloxone infusion in LMAN also did not reveal any changes when these measures were compared to controls. However, an analysis of the CV of individual syllables revealed that there was a significant change in the goodness of pitch in harmonic stacks during naloxone infusions versus controls (RM ANOVA; *F* = 5.45; df = 2, 8; *P* = 0.032). This difference stemmed from a significant decrease in the CV of goodness of pitch in harmonic stack syllables during infusions of 200 ng/ml naloxone (8.81 ± 0.57) in LMAN versus controls (11.90 ± 1.12).

#### Changes in the Mean Accuracy of Motifs Resulted From Changes in the Acoustic Features of Individual Syllables

**FIGURE 8:**
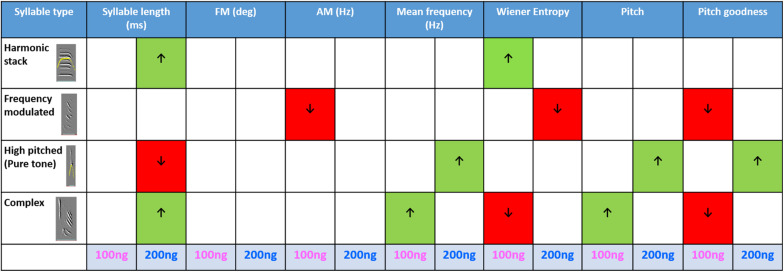
Summary of the effects of blocking ORs in LMAN on the acoustic features of various syllable types: The table shows acoustic features during infusions of 100 and 200 ng/ml naloxone compared to aCSF (One Way ANOVA on ranks, Dunn’s *Post hoc* test). Arrows pointing upward and green cells indicate an increase whereas arrows pointing downward and red cells indicate a decrease in each case. The *P*-value for significance was set to <0.01.

To further investigate which syllable types contributed to the decrease in the mean accuracy of motifs while ORs were blocked in LMAN, we analyzed the acoustic features of various syllable types of experimental birds. The four most commonly occurring syllable types sung by the birds used in our analysis were harmonic stacks, frequency-modulated syllables, high-pitched syllables and complex syllables. A Kruskal–Wallis One Way ANOVA on Ranks test revealed that different syllable types were differently affected by 100 ng/ml and 200 ng/ml of naloxone compared to those sung during aCSF infusion in LMAN (Figure 8; see Supplementary Tables 3–6 for details). We observed that in case of the harmonic syllables, the syllable length increased significantly with infusions of 200 ng/ml naloxone compared to controls. Amongst spectral features, the only change observed was a significant increase in Wiener entropy during infusions of 100 ng/ml naloxone, suggesting that they became noisier when ORs were blocked in LMAN. Infusions of 100 ng/ml naloxone in LMAN also led to significant decreases in AM and pitch goodness of frequency-modulated syllables, whereas the Wiener entropy of these syllables decreased during infusions of 200 ng/ml naloxone versus controls. These results suggest that the harmonic structure of frequency-modulated syllables decreased and they became less noisy as a result of decreases in OR modulation. We also found that in the case of high-pitched syllables, whereas infusions of 200 ng/ml naloxone led to significant decreases in the length, there were significant increases in the mean frequency, pitch and pitch goodness with this dose of naloxone. In complex syllables (combinations of high-pitched notes followed by frequency-modulated notes), there were significant increases in the mean frequency and pitch during infusions of 100 ng/ml naloxone in LMAN. These changes were accompanied by a significant decrease in pitch goodness at the same dose. Furthermore, infusions of 200 ng/ml naloxone in LMAN led to a significant increase in the length of these syllables.

Taken together, these results suggest that blocking ORs in LMAN lead to changes in the acoustic features of individual syllables, which are reflected in the significant changes in mean accuracy at the level of motifs. However, since there were significant increases and decreases in the acoustic features of different syllable types with both doses of naloxone (100 and 200 ng/ml) used in our study, they were not reflected at the level of motifs (Supplementary Table 2) or in % similarity scores (Figure 7C) which measure similarity at global (motif) levels. Furthermore, the length of individual syllables appeared to be affected only by the 200 ng/ml dose of naloxone whereas spectral features were differentially affected depending on the dose of naloxone infused in LMAN.

## Discussion

In the present study, we confirmed that almost all LMAN neurons express μ-ORs, including magnocellular neurons which were negative for GAD67 and project to Area X and RA (Nottebohm et al., 1976; Nixdorf-Bergweiler et al., 1995; Vates and Nottebohm, 1995) and smaller interneurons which also express GAD67 (Fan et al., 2018; Mukherjee and Caroni, 2018). These results suggest that the μ-OR system may modulate the microcircuitry within LMAN as well as the activity of neurons in Area X via its downstream projections. Since projection neurons in LMAN also express δ-ORs (Khurshid et al., 2009; Singh and Iyengar, unpublished) and naloxone is a non-selective antagonist with greater affinity for μ- than for δ-ORs (Magnan et al., 1982; Deviche, 1997), it is possible that both OR subtypes can modulate the activity of LMAN neurons. However, higher levels of μ-ORs are expressed than δ-ORs in different song control regions including LMAN (Khurshid et al., 2009), suggesting that they may play a greater role in mediating the behavioral effects of opioid modulation. Since the same LMANcore neurons which project to Area X also project directly to RA (Bottjer et al., 1984; Johnson et al., 1995; Bottjer and Altenau, 2010), opioid modulation via LMAN may have dual effects on RA and the acoustic properties of song.

### Altering Opioid Modulation in LMAN May Affect the Motivation of Males to Sing to Females via Its Downstream Projections to the Reward Pathways

We found that experimental birds initiated fewer bouts and sang fewer introductory notes during naloxone infusion in LMAN compared to controls, although these differences were not significant. Furthermore, there were no changes in the number of INs or motifs per bout. Similar results, that is, no changes in the IN to motif ratio per bout were also obtained when LMAN was inactivated by infusions of the GABA(A) receptor agonist muscimol (Stepanek and Doupe, 2010), although this study did not analyze changes in the number of FD bouts. Taken together, these results suggest that ratio of INs or motifs to bouts is not affected by opioid or GABAergic receptors. We also found that despite the significant decrease in FD singing during infusions of different doses of naloxone in LMAN, there were no differences in the levels of visual attention paid by the males toward females. Kao and Brainard (2006) had reported that levels of visual attention or the number of INs and motifs did not change during the production of FD bouts before and after lesions of LMAN. Our results therefore suggest that blocking ORs in LMAN specifically affected the motivation to sing FD motifs.

Recent findings in rodents suggest that the modulation of μ-ORs expressed in the cortex may affect motivation, reward and motor activity (Baldo, 2016). The basal ganglia in mammals are known to be involved in various aspects of motor behavior including vocalization and cognitive functions such as reward, motivation (Brown et al., 1997; Cardinal et al., 2002; Reep et al., 2003; Haruno et al., 2004; Shohamy et al., 2004; Delgado, 2007), and emotion-driven changes in speech (reviewed in Ziegler and Ackermann, 2017). Therefore, it is likely that the motivation to sing FD songs might be mediated via LMAN (cortical/pallial) projections to Area X (a basal ganglia homologue). Blocking ORs in LMAN may alter the balance between excitatory projection neurons and GABAergic interneurons within this region, further altering levels of neural activity in Area X, which receives excitatory input from LMAN and projects indirectly to VTA-SNc which is involved in reward and motivation (Yanagihara and Hessler, 2006; Hara et al., 2007; Huang and Hessler, 2008) via VP (Gale et al., 2008). Interestingly, earlier studies (Riters and Alger, 2004; Riters, 2012) have described reciprocal projections between VTA and mPOA, which is known to affect FD singing (Kelm-Nelson et al., 2013). Since VTA lies downstream to LMAN and Area X, it is possible that opioid modulation in LMAN could affect the motivation to sing via projections between VTA and mPOA.

### The Levels of Neurotransmitters in LMAN Are Not Affected by Blocking OR Modulation

There were no significant changes in the levels of glutamate, GABA or dopamine in LMAN during naloxone infusions versus controls. The lack of significant changes in DA levels in LMAN may have resulted from the fact that its core region (which projects to Area X) does not receive high levels of dopaminergic innervation in zebra finches (Sakaguchi and Saito, 1989; Bottjer, 1993; Soha et al., 1996; Harding et al., 1998; Singh and Iyengar, 2019). Earlier studies have shown that unlike DA, there is little leakage of either glutamate or GABA away from synapses and into extracellular fluid due to the presence of tight junctions and efficient reuptake mechanisms (Drew et al., 2004; Watson et al., 2006). Therefore, we cannot rule out the possibility that blocking ORs in LMAN may have led to small changes in the levels of glutamate and/or GABA in this nucleus, which were undetectable.

### Changes in OR Modulation in LMAN Lead to Alterations in the Acoustic Features of FD Song

We found that amongst various acoustic features, the only two which changed significantly following infusions of naloxone in LMAN were motif length and AM of FD songs. An analysis of similarity scores across motifs sung during vehicle or naloxone infusions in LMAN also demonstrated changes in mean accuracy, which depends on fine-grain changes in spectral features at the level of individual syllables. It is possible that these changes may have resulted from direct connections between LMAN and the premotor cortical region RA, which controls the spectral features of individual syllables (Vicario, 1991; Vu et al., 1994; Kao et al., 2005; Olveczky et al., 2005; Ashmore et al., 2008a). Additionally, altered activity patterns in LMAN may have led to changes in the activity of the Area X → DLM → LMAN → RA loop, thus changing the acoustic properties of FD song (Leblois et al., 2010; Leblois and Perkel, 2012; Miller et al., 2015; Kojima et al., 2018; Kumar et al., 2019).

We also found that OR modulation in LMAN and Area X appear to have contrasting effects on the spectral properties of song (Heston et al., 2018). In an earlier study, blocking ORs in Area X led to significant decreases in FM, AM and pitch goodness, and a significant increase in pitch, whereas Wiener entropy and mean frequency of motifs was not affected (Kumar et al., 2019). In the present study, the same manipulation in LMAN led to significant changes in motif length and AM, without affecting other features.

At the level of individual syllables, we had earlier shown that except for frequency-modulated syllables, the other syllable types (harmonic stacks, high-pitched, and complex) were altered by blocking ORs in Area X. Furthermore, blocking ORs in Area X had the greatest effect on the acoustic properties of harmonic stacks (Kumar et al., 2019). In contrast, blocking ORs in LMAN led to changes in the spectral features of all syllable types. Whereas three or more acoustic properties were affected by altering opioid modulation in LMAN in the case of frequency-modulated, high-pitched and complex syllables, only two features (increases in length and noisiness) were observed for harmonic stacks. These results suggest that altering opioid modulation in LMAN and Area X had different effects on the acoustic properties of individual syllables.

In our study, we did not find changes in the variability of most of the acoustic features of motifs or syllables. The only difference was a significant decrease in variability of harmonic stack syllables during infusions of 200 ng/ml naloxone versus controls. Taken together, our results suggest that changes in neural activity in LMAN caused by altered opioid modulation may not have been sufficient to lead to changes in the overall variability of FD songs at the global level but may have very subtle effects at fine-grain levels. Alternatively, it is possible that the variability in song and acoustic features of syllables via LMAN may be controlled by modulators other than the endogenous opioid system. Furthermore, it is possible that spectral variability in the context of FD song is vested mainly in GPi output neurons of Area X and only partly depends on cortical input from LMAN neurons (Woolley et al., 2014; Kojima et al., 2018).

### Blocking ORs in LMAN May Have Affected the Temporal Features of FD Motifs via Indirect Projections Between LMAN to HVC

Blocking ORs in LMAN also led to increases in the temporal properties (motif and syllable lengths) of FD songs. The timing and sequence of individual syllables in motifs are controlled by HVC (Hahnloser et al., 2003; Leonardo and Fee, 2005). There are at least three possible routes by which neural activity in LMAN can reach HVC. The first pathway connects LMAN→RA→DM→Uva→HVC (Wild, 2004; Ashmore et al., 2008b). It includes direct projections from LMAN to RA, which in turn, projects to brainstem respiratory centers (DM; dorsomedial nucleus of the intercollicular complex; Margoliash and Konishi, 1985; Wild, 1993, 1997). Neurons in DM project to Uva [uvaeform nucleus of the thalamus; (Wild, 1993; Ashmore et al., 2005)] of both hemispheres which is one of the main inputs to HVC. Furthermore, Uva projects to NIf [Nucleus interfacialis; Nottebohm et al. (1982)] and Av [nucleus Avalanche; (Nottebohm et al., 1982)], both of which integrate auditory information and also project to HVC (Nottebohm et al., 1982; Vicario, 1991; Wild, 1993; Akutagawa and Konishi, 2010). The two other circuits connecting LMAN and HVC include the LMAN→Area X→VP→A11→HVC circuit (Appeltants et al., 2000; Hamaguchi and Mooney, 2012) and the LMAN→Area X→VP→VTA→HVC circuit (Appeltants et al., 2000). Both VTA and A11 are dopaminergic and the pathway through A11 is known to drive propagation of activity rapidly at much shorter (5–15 ms) latencies and real time changes in song structure (Hamaguchi and Mooney, 2012). Thus, altering opioid modulation within LMAN may have altered the temporal features of FD song via indirect projections to HVC.

## Conclusion

Blocking ORs in LMAN lead to a decrease in the number of FD motifs, without affecting visual attention toward females. Whereas these manipulations did not have major effects on the acoustic properties of motifs, fine-grain changes (at the level of individual syllables) were observed. Despite the fact that they are directly connected, opioid modulation in LMAN and Area X appear to affect the FD songs in contrasting ways. Our findings also suggest that opioid modulation of cortico-striatal pathway can influence not only the acoustic properties of FD song but also the motivation to sing. Given that altering opioid modulation in different components of the AFP leads to changes in the acoustic properties of their songs, zebra finches are an excellent model system to study the effects of opioid abuse on the production of vocalizations.

## Data Availability Statement

The raw data supporting the conclusions of this article will be made available by the authors, without undue reservation.

## Ethics Statement

The animal study was reviewed and approved by Institutional Animal Ethics Committee at the National Brain Research Centre, Manesar (NBRC), India.

## Author Contributions

SK and SI designed the experiments, interpreted data, and drafted the manuscript. SK and AM performed surgeries and experiments. MK and SK standardized and performed procedures for immunoblots. SK and AP performed immunohistochemistry and UD and SS assisted with surgeries and the analysis of songs. AD and VA helped in determining the exact coordinates of LMAN using electrophysiology in preliminary experiments prior to cannula implants. SI conceptualized and supervised the study and corrected the final draft of the manuscript. All authors contributed to scientific discussions regarding the data and approved the final manuscript.

## Conflict of Interest

The authors declare that the research was conducted in the absence of any commercial or financial relationships that could be construed as a potential conflict of interest.

## Supplementary Material

FIGURE S1 Validation for the specificity of the anti-GAD67 antibody in zebra finch brain tissue (Abcam, ab26116, made in mouse). Bands in the range of 180 and 140 kDa, corresponding to trimers and dimers of GAD67 either with itself or with GAD65 (Kanaani et al., 1999). Smaller fractions in the range of 110, 50, and 31 kDa were also detected as observed in rodents using the same antibody [Szabo et al. (1994), Benitez et al. (2014), and Yu et al. (2016)], which may represent truncated forms of the dimers and monomers of GAD67 (Battaglioli et al., 2005; Sha et al., 2005).

FIGURE S2 Self-similarity scores for control trials (aCSF) and trials with naloxone treatment (One Way Repeated Measures ANOVA). Changes in self-similarity reflect changes in the variability within each group. **(A)** The % similarity (at motif level) did not change for any group during naloxone infusions in LMAN. **(B)** The % mean accuracy scores which measures changes at the level of individual syllables also did not change significantly for any group during infusions of naloxone in LMAN.
