# Supplementary figures and images for: Blocking Opioid Receptors in a Songbird Cortical Region Modulates the Acoustic Features and Levels of Female-Directed Singing
Source: Front Neurosci. 2020 Sep 17;14:554094. doi: 10.3389/fnins.2020.554094 (PMC7533562; doi:10.3389/fnins.2020.554094)

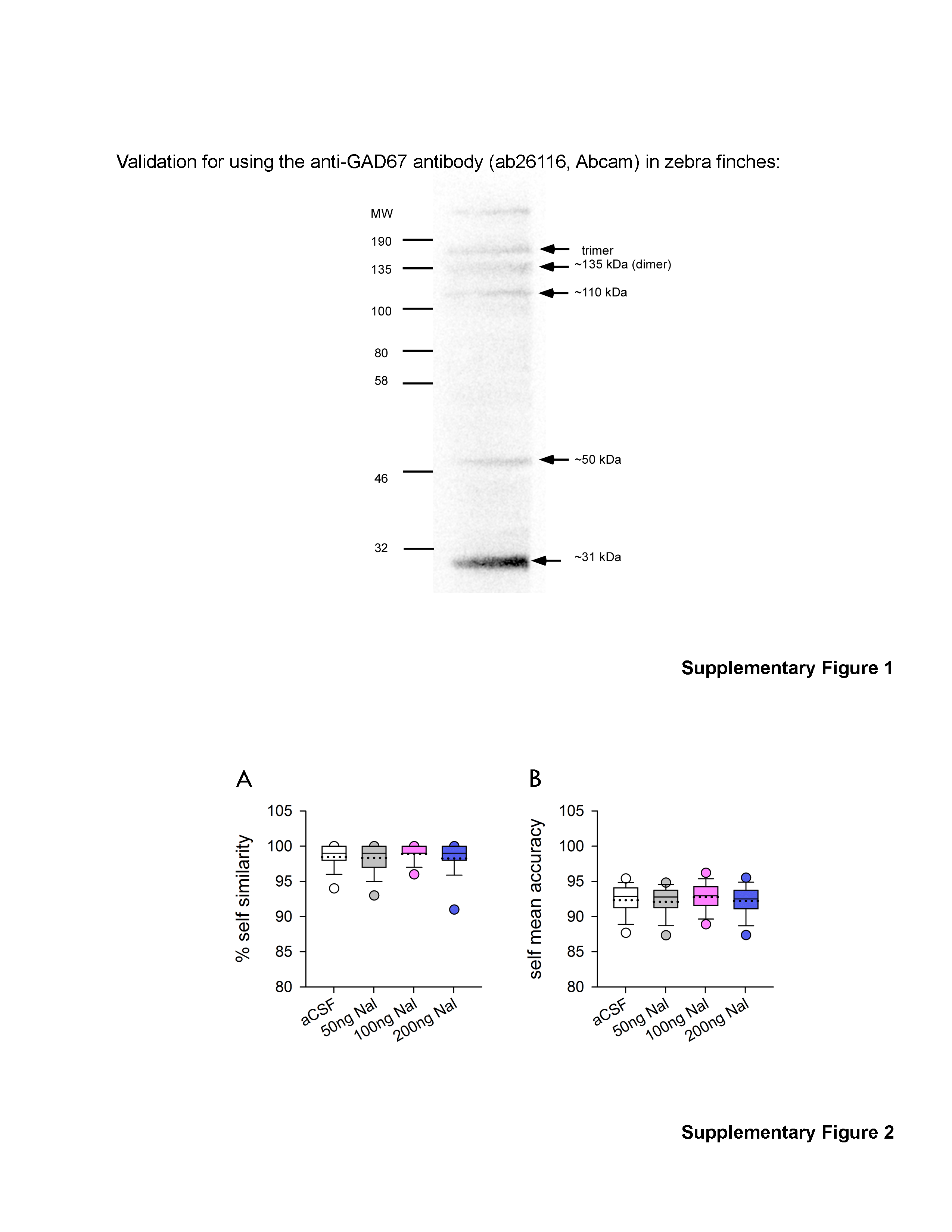

Supplement: FIGURE S1 — Validation for the specificity of the anti-GAD67 antibody in zebra finch brain tissue (Abcam, ab26116, made in mouse). Bands in the range of 180 and 140 kDa, corresponding to trimers and dimers of GAD67 either with itself or with GAD65 (Kanaani et al., 1999). Smaller fractions in the range of 110, 50, and 31 kDa were also detected as observed in rodents using the same antibody [Szabo et al. (1994), Benitez et al. (2014), and Yu et al. (2016)], which may represent truncated forms of the dimers and monomers of GAD67 (Battaglioli et al., 2005; Sha et al., 2005). [file Image_1.TIF]
