# Supplementary material for: Blocking Opioid Receptors in a Songbird Cortical Region Modulates the Acoustic Features and Levels of Female-Directed Singing
Source: Front Neurosci. 2020 Sep 17;14:554094. doi: 10.3389/fnins.2020.554094 (PMC7533562; doi:10.3389/fnins.2020.554094)
Supplement: Supplementary file 2 [file Table_1.docx]

| **Suppl. Table 1:** Comparison of the acoustic features of motifs on different days of the experiments (Kruskal-Wallis One Way ANOVA on Ranks) | | | | |
| --- | --- | --- | --- | --- |
| **Acoustic features** | **Experiment** | **H value** | **df** | **P-value** |
| Motif Length | aCSF | 1.971 | 11 | 1.00 |
|  | 100ng Nal | 0.805 | 3 | 0.85 |
|  | 200ng Nal | 0.846 | 3 | 0.84 |
| ISI | aCSF | 1.586 | 11 | 1.00 |
|  | 100ng Nal | 0.157 | 3 | 0.98 |
|  | 200ng Nal | 1.849 | 3 | 0.60 |
| Pitch | aCSF | 3.037 | 11 | 0.99 |
|  | 100ng Nal | 0.333 | 3 | 0.95 |
|  | 200ng Nal | 2.037 | 3 | 0.57 |
| FM | aCSF | 4.263 | 11 | 0.96 |
|  | 100ng Nal | 2.924 | 3 | 0.40 |
|  | 200ng Nal | 1.157 | 3 | 0.76 |
| AM | aCSF | 6.582 | 11 | 0.83 |
|  | 100ng Nal | 0.505 | 3 | 0.92 |
|  | 200ng Nal | 0.253 | 3 | 0.97 |
| Entropy | aCSF | 5.714 | 11 | 0.89 |
|  | 100ng Nal | 1.338 | 3 | 0.72 |
|  | 200ng Nal | 0.433 | 3 | 0.93 |
| Pitch Goodness | aCSF | 1.159 | 11 | 1.00 |
|  | 100ng Nal | 0.367 | 3 | 0.95 |
|  | 200ng Nal | 0.668 | 3 | 0.88 |
| Mean Frequency | aCSF | 9.713 | 11 | 0.56 |
|  | 100ng Nal | 1.381 | 3 | 0.71 |
|  | 200ng Nal | 0.121 | 3 | 0.99 |

The acoustic features of motifs did not change over the course of experiments

within groups either during control (aCSF infusion: 1^st^ to 12^th^ day) or within

treatments (100 or 200ng/ml naloxone infusion: 1^st^ to 4^th^ day).

| **Suppl. Table 2:** Comparison of acoustic features of motifs following infusions of different doses of naloxone in LMAN (Kruskal-Wallis One Way ANOVA on Ranks) | | | | | | |
| --- | --- | --- | --- | --- | --- | --- |
| **Acoustic features** | **Experiment** | **n** | **Median** | **P-value Dunn's Multiple comparison** | **Main effects** | |
|  |  |  |  |  | **H value** | **P-value** |
| Motif Length | aCSF | 414 | 545.36 |  | 9.50 | 0.023 |
|  | 50ng Nal | 87 | 556.24 |  |  |  |
|  | 100ng Nal | 81 | 523.60 | 0.023 (200ng Nal vs 100ng Nal) |  |  |
|  | 200ng Nal | 89 | 543.32 |  |  |  |
| Syllable Length | aCSF | 414 | 40.12 |  | 1.86 | 0.602 |
|  | 50ng Nal | 87 | 39.78 |  |  |  |
|  | 100ng Nal | 81 | 38.99 |  |  |  |
|  | 200ng Nal | 89 | 42.16 |  |  |  |
| Pitch | aCSF | 414 | 1423.50 |  | 4.77 | 0.189 |
|  | 50ng Nal | 87 | 1277.00 |  |  |  |
|  | 100ng Nal | 81 | 1422.00 |  |  |  |
|  | 200ng Nal | 89 | 1262.00 |  |  |  |
| FM | aCSF | 414 | 48.10 |  | 3.55 | 0.314 |
|  | 50ng Nal | 87 | 47.60 |  |  |  |
|  | 100ng Nal | 81 | 47.50 |  |  |  |
|  | 200ng Nal | 89 | 47.40 |  |  |  |
| AM | aCSF | 414 | 0.0038 |  | 10.01 | 0.018 |
|  | 50ng Nal | 87 | 0.0039 |  |  |  |
|  | 100ng Nal | 81 | 0.0037 | 0.021 (200ng Nal vs 100ng Nal) |  |  |
|  | 200ng Nal | 89 | 0.0039 |  |  |  |
| Entropy | aCSF | 414 | -2.18 |  | 6.38 | 0.094 |
|  | 50ng Nal | 87 | -2.32 |  |  |  |
|  | 100ng Nal | 81 | -2.04 |  |  |  |
|  | 200ng Nal | 89 | -2.08 |  |  |  |
| Pitch Goodness | aCSF | 414 | 648.35 |  | 2.74 | 0.434 |
|  | 50ng Nal | 87 | 625.60 |  |  |  |
|  | 100ng Nal | 81 | 669.80 |  |  |  |
|  | 200ng Nal | 89 | 659.30 |  |  |  |
| Mean Frequency | aCSF | 414 | 3808.00 |  | 4.26 | 0.234 |
|  | 50ng Nal | 87 | 3770.00 |  |  |  |
|  | 100ng Nal | 81 | 3734.00 |  |  |  |
|  | 200ng Nal | 89 | 3804.00 |  |  |  |

| **Suppl. Table 3:** Comparison of acoustic features of harmonic syllables (Kruskal-Wallis One Way ANOVA on Ranks) | | | | | | |
| --- | --- | --- | --- | --- | --- | --- |
| **Acoustic features** | **Experiment** | **n** | **Median** | **P-value compared to aCSF (Dunn)** | **Main effects** | |
|  |  |  |  |  | **H value** | **P-value** |
| Syllable Duration | aCSF | 361 | 103.36 |  |  |  |
|  | 100ng Nal | 96 | 100.64 | 0.431 | 11.42 | **0.003** |
|  | 200ng Nal | 150 | 110.16 | **0.012** |  |  |
| Pitch | aCSF | 361 | 752.00 |  |  |  |
|  | 100ng Nal | 96 | 748.00 |  | 6.88 | 0.032 |
|  | 200ng Nal | 150 | 752.50 |  |  |  |
| FM | aCSF | 361 | 23.80 |  |  |  |
|  | 100ng Nal | 96 | 22.90 |  | 4.30 | 0.117 |
|  | 200ng Nal | 150 | 22.30 |  |  |  |
| AM | aCSF | 361 | 0.0114 |  |  |  |
|  | 100ng Nal | 96 | 0.0108 |  | 4.81 | 0.09 |
|  | 200ng Nal | 150 | 0.0127 |  |  |  |
| Entropy | aCSF | 361 | -2.89 |  |  |  |
|  | 100ng Nal | 96 | -2.78 | **<0.001** | 22.02 | **<0.001** |
|  | 200ng Nal | 150 | -2.93 | 1.000 |  |  |
| Pitch Goodness | aCSF | 361 | 921.80 |  |  |  |
|  | 100ng Nal | 96 | 925.70 |  | 2.74 | 0.254 |
|  | 200ng Nal | 150 | 353.90 |  |  |  |
| Mean Frequency | aCSF | 361 | 3910.00 |  |  |  |
|  | 100ng Nal | 96 | 3891.50 |  | 1.92 | 0.382 |
|  | 200ng Nal | 150 | 3928.50 |  |  |  |

| **Suppl. Table 4**: Comparison of acoustic features of frequency-modulated syllables (Kruskal-Wallis One Way ANOVA on Ranks) | | | | | | |
| --- | --- | --- | --- | --- | --- | --- |
| **Acoustic features** | **Experiment** | **n** | **Median** | **P-value compared to aCSF (Dunn)** | **Main effects** | |
|  |  |  |  |  | **H value** | **P-value** |
| Syllable Duration | aCSF | 907 | 66.64 |  |  |  |
|  | 100ng Nal | 260 | 82.96 |  | 1.51 | 0.469 |
|  | 200ng Nal | 337 | 67.32 |  |  |  |
| Pitch | aCSF | 907 | 1081.00 |  |  |  |
|  | 100ng Nal | 260 | 957.50 |  | 6.79 | 0.033 |
|  | 200ng Nal | 337 | 1009.00 |  |  |  |
| FM | aCSF | 907 | 52.50 |  |  |  |
|  | 100ng Nal | 260 | 52.60 |  | 2.15 | 0.341 |
|  | 200ng Nal | 337 | 52.70 |  |  |  |
| AM | aCSF | 907 | 0.0176 |  |  |  |
|  | 100ng Nal | 260 | 0.0155 | **<0.001** | 17.31 | **<0.001** |
|  | 200ng Nal | 337 | 0.0163 | 0.166 |  |  |
| Entropy | aCSF | 907 | -1.79 |  |  |  |
|  | 100ng Nal | 260 | -1.84 |  | 18.39 | **<0.001** |
|  | 200ng Nal | 337 | -1.93 | **<0.001** |  |  |
| Pitch Goodness | aCSF | 907 | 723.10 |  |  |  |
|  | 100ng Nal | 260 | 594.00 | **<0.001** | 22.67 | **<0.001** |
|  | 200ng Nal | 337 | 847.70 | 0.148 |  |  |
| Mean Frequency | aCSF | 907 | 3902.00 |  |  |  |
|  | 100ng Nal | 260 | 3974.50 |  | 1.25 | 0.535 |
|  | 200ng Nal | 337 | 3866.00 |  |  |  |

| **Suppl. Table 5**: Comparison of acoustic features of high-pitched syllables (Kruskal-Wallis One Way ANOVA on Ranks) | | | | | | |
| --- | --- | --- | --- | --- | --- | --- |
| **Acoustic features** | **Experiment** | **n** | **Median** | **P-value compared to aCSF (Dunn)** | **Main effects** | |
|  |  |  |  |  | **H value** | **P-value** |
| Syllable Duration | aCSF | 534 | 31.28 |  |  |  |
|  | 100ng Nal | 109 | 29.92 | 0.933 | 16.00 | **<0.001** |
|  | 200ng Nal | 174 | 23.12 | **<0.001** |  |  |
| Pitch | aCSF | 534 | 4711.00 |  |  |  |
|  | 100ng Nal | 109 | 4487.00 | 1.000 | 17.88 | **<0.001** |
|  | 200ng Nal | 174 | 5089.00 | **<0.001** |  |  |
| FM | aCSF | 534 | 68.70 |  |  |  |
|  | 100ng Nal | 109 | 67.90 |  | 1.44 | 0.486 |
|  | 200ng Nal | 174 | 68.10 |  |  |  |
| AM | aCSF | 534 | 0.0692 |  |  |  |
|  | 100ng Nal | 109 | 0.0687 |  | 2.97 | 0.226 |
|  | 200ng Nal | 174 | 0.0708 |  |  |  |
| Entropy | aCSF | 534 | -2.97 |  |  |  |
|  | 100ng Nal | 109 | -3.10 |  | 5.11 | 0.078 |
|  | 200ng Nal | 174 | -2.56 |  |  |  |
| Pitch Goodness | aCSF | 534 | 510.90 |  |  |  |
|  | 100ng Nal | 109 | 500.40 | 0.616 | 23.89 | **<0.001** |
|  | 200ng Nal | 174 | 567.65 | **<0.001** |  |  |
| Mean Frequency | aCSF | 534 | 5285.00 |  |  |  |
|  | 100ng Nal | 109 | 5330.00 | 1.000 |  | **0.011** |
|  | 200ng Nal | 174 | 5324.50 | **0.006** |  |  |

| **Suppl. Table 6**: Comparison of acoustic features of complex syllables (Kruskal-Wallis One Way ANOVA on Ranks) | | | | | | |
| --- | --- | --- | --- | --- | --- | --- |
| **Acoustic features** | **Experiment** | **n** | **Median** | **P-value compared to aCSF (Dunn)** | **Main effects** | |
|  |  |  |  |  | **H value** | **P-value** |
| Syllable Duration | aCSF | 316 | 85.68 |  |  |  |
|  | 100ng Nal | 89 | 87.04 | 0.344 | ﻿11.706 | **0.003** |
|  | 200ng Nal | 62 | 89.76 | **0.010** |  |  |
| Pitch | aCSF | 316 | 3781.50 |  |  |  |
|  | 100ng Nal | 89 | 4127.00 | **<0.001** | ﻿13.544 | **0.001** |
|  | 200ng Nal | 62 | 3856.00 | 1.000 |  |  |
| FM | aCSF | 316 | 48.05 |  |  |  |
|  | 100ng Nal | 89 | 49.40 |  | ﻿0.676 | 0.713 |
|  | 200ng Nal | 62 | 49.40 |  |  |  |
| AM | aCSF | 316 | 0.0266 |  |  |  |
|  | 100ng Nal | 89 | 0.0261 |  | ﻿6.756 | ﻿0.034 |
|  | 200ng Nal | 62 | 0.0274 |  |  |  |
| Entropy | aCSF | 316 | -2.25 |  |  |  |
|  | 100ng Nal | 89 | -2.41 | **<0.001** | ﻿20.639 | **<0.001** |
|  | 200ng Nal | 62 | -2.28 | 0.732 |  |  |
| Pitch Goodness | aCSF | 316 | 534.40 |  |  |  |
|  | 100ng Nal | 89 | 476.10 | **0.001** | ﻿13.228 | **﻿0.001** |
|  | 200ng Nal | 62 | 521.55 | 0.118 |  |  |
| Mean Frequency | aCSF | 316 | 3794.50 |  |  |  |
|  | 100ng Nal | 89 | 3868.00 | **0.001** | ﻿11.864 | **0.003** |
|  | 200ng Nal | 62 | 3777.00 | 1.000 |  |  |
